# Who Said What: A Multi-Country Content Analysis of European Health Organisations’ COVID-19 Social Media Communication

**DOI:** 10.3389/ijph.2022.1604973

**Published:** 2022-09-22

**Authors:** Kamyar Kompani, Michael J. Deml, Farnaz Mahdavian, Olena Koval, Sanjana Arora, Hilda Broqvist

**Affiliations:** ^1^ Department of Sociology, Institute of Sociological Research, University of Geneva, Geneva, Switzerland; ^2^ Division of Social and Behavioural Sciences, School of Public Health and Family Medicine, University of Cape Town, Cape Town, South Africa; ^3^ DIALOGIK, Stuttgart, Germany; ^4^ Department of Safety, Economics and Planning, University of Stavanger, Stavanger, Norway; ^5^ Department of Humanities and Social Sciences, Mid Sweden University, Östersund, Sweden

**Keywords:** social media, COVID-19, risk communication, Switzerland, Germany, Norway, Sweden, UK

## Abstract

**Objectives:** As a risk communication tool, social media was mobilised at an unprecedented level during the COVID-19 pandemic. This study examined health authorities’ risk communication on social media in response to the pandemic in 2020.

**Methods:** We analysed 1,633 COVID-19-related posts from 15 social media accounts managed by official health authorities in Germany, Norway, Sweden, Switzerland, and the United Kingdom.

**Results:** The rate at which the authorities posted about COVID-19 on social media fluctuated throughout 2020. Each account’s posting frequency peaked between March and May 2020, before dropping considerably during the summer. The messages that the organisations focused on also varied throughout the year but covered most risk communication guidelines. Yet, our analysis highlighted themes that were communicated infrequently, such as long COVID or exercising during the pandemic.

**Conclusion:** With more individuals now following health authorities on social media, platforms such as Instagram hold great potential for future risk communication campaigns and strategies.

## Introduction

On 11 March 2020, the World Health Organization (WHO) designated the COVID-19 outbreak as a global pandemic. Meanwhile, health authorities worldwide, including risk communicators, were racing to implement their response plans. Research has shown that risk communication is integral to effective risk management during a pandemic [1,2,3]. Risk communication, which emerged as a specialised field in the 1980s, is defined by the WHO as the “real-time exchange of information, advice and opinions between experts, community leaders, officials and the people who are at risk and is an integral part of any emergency response” [4].

The field of risk communication has continuously evolved over the past few decades. At first, risk communication was conceived as one-sided. Experts were tasked with sharing information with the public [5]. Only experts were thought to have access to scientific information, and the public was seen as a passive recipient [5]. Risk communication was therefore considered a one-way approach based on basic assumptions about the public [6]. Over the years, risk communicators started to emphasise the “interactive process of information exchange”, taking into consideration the public’s perception of risk [7]. Risk communication is now considered a two-way street with an active audience that contributes to risk and crisis management [5,5,6].

One of the main models of risk communication is the Centers for Disease Control and Prevention’s (CDC’s) Crisis and Emergency Risk Communication (CERC). The CERC model, which is based on lessons learned from past public health emergencies and research from different scientific fields, divides health crises into five stages and advances six primary recommendations: to communicate as early, correctly and credibly as possible, while also expressing empathy, promoting concrete actions that people can take, and showing respect by promoting collaboration and rapport [8].

Previously, health communicators relied on traditional media and physical mediums (e.g., posters and leaflets), and, more recently, websites, to reach the public [9]. The most-popular social media platforms, such as Facebook and Instagram, were launched less than 2 decades ago. As such, they’ve been mobilised during a limited number of health crises, most notably, in response to A(H1N1) in 2009 and Ebola in 2014 [10,11], which has also restricted the opportunities that researchers have had to study health authorities’ risk communication on social media. This is clearly no longer the case with the ongoing COVID-19 pandemic.

In adherence with WHO risk communication guidelines that recommend the use of social media alongside traditional mediums [4], health authorities worldwide disseminated their COVID-19 messages on social media during the pandemic. The WHO suggests that social media can help to engage with the public, create situational awareness, and facilitate local-level responses [4].

Often inactive on social media—except on Twitter—prior to the pandemic, European health authorities saw their Facebook, Instagram and Twitter accounts grow rapidly in the early months of the pandemic. For example, the Facebook accounts of the National Health Service United Kingdom (NHSUK) and the German Federal Ministry of Health (BMG) each gained over 500,000 followers between February and April 2020 [13]. On average, the Facebook accounts analysed in this study increased their number of followers by sixfold during the same period, while the Instagram accounts grew by double that rate [13].

In this study, we analysed the German, Norwegian, Swedish, Swiss, and United Kingdom (UK) health authorities’ COVID-19-related communication on Facebook, Instagram and Twitter. As five Western European countries that share numerous traits, such as being ranked in the top 30 wealthiest countries based on their GDP and having similar social media landscapes [14, 15, 16, 17, 18, 19], they differ in myriad ways, including in the organisation of their health sectors and their relationship with the EU, as well as how they responded to the pandemic in 2020, highlighting “the country-specific nature of pandemic responses from its beginnings” [20]. Germany and Switzerland pursued a “federal approach with strong federal-level authority exercised at the onset of the pandemic, followed by delegation of decision-making to the countries’ many autonomous Länder and cantons (respectively)”. The UK had a “devolved response” where England, Northern Ireland, Scotland, and Wales coordinated initially, but then eased up their restrictions at different rates (Ibid). Finally, Sweden largely took a “hands-off approach” and relied on “voluntary adherence to COVID-19 mitigation measures”, while Norway “enacted limited legislation for decentralised responses throughout its 11 regions” [20].

The five countries also varied in the combination of public organisations—in addition to various levels of government—that communicated about COVID-19 and the pandemic to the public. In Germany, the main communication sources were two health organisations, the Federal Ministry of Health (BMG) and the Robert Koch Institute (RKI) [21]. In Norway, the Norwegian Directorate of Health (NDH) and the Norwegian Institute of Public Health (NIPH) were joined by the Ministry of Justice and the Ministry of Health and Care Services [21]. In Sweden, alongside the Public Health Agency of Sweden (PHAS) and the National Board of Health, the Welfare and the Swedish Civil Contingencies Agency also communicated about COVID-19. In Switzerland, the Federal Office of Public Health (FOPH) was responsible for communicating about the virus and pandemic. Finally, in the UK, both the National Health Service (NHS) and nation-specific health bodies under the Department of Health and Social Care were involved in the communication [21].

## Methods

We applied a content analysis to study what health authorities from each of the five countries communicated about COVID-19 on social media in 2020. The unit of analysis is the “post” (i.e., Facebook post, Instagram post and tweets/retweets) and includes the text featured in the caption as well as the accompanying image (typically a poster or infographic) or video. For each account, we randomly coded between 100 and 150 posts related to COVID-19 published in 2020. Some accounts had fewer than 100 COVID-19-related posts in total (see Table 1 for a breakdown of each account). In all cases, we reached a point of saturation during the coding process, with similar messages—often the exact same post—emerging repeatedly. This was particularly the case on Twitter, where it is customary to reshare the same post multiple times (not including retweets). We collected Facebook and Instagram posts with CrowdTangle, a public insights tool owned and operated by Facebook, and obtained tweets with NCapture and NVivo, before exporting them as Excel sheets. Our study sample contains 1,633 posts from 15 accounts, including twitter retweets.

**TABLE 1:** Breakdown of dataset per country and account (Geneva, Switzerland. 2022).

Account	Total Posts in 2020	Number of Posts Analysed (% of total)	Number of Posts Related to Covid-19 and Coded (% of total posts analysed)
Germany BMG Facebook	661	106 (16%)	100 (94%)
Germany BMG Instagram	315	108 (34%)	100 (93%)
Germany RKI Twitter	315	123 (39%)	97 (79%)
Norway NDH Facebook	102	102 (100%)	84 (82%)
Norway NDH Twitter	38	38 (100%)	37 (97%)
Norway NIPH Facebook	110	110 (100%)	82 (75%)
Norway NIPH Twitter	290	228 (78%)	150 (66%)
Sweden PHAS Facebook	89	89 (100%)	74 (83%)
Sweden PHAS Twitter	280	178 (64%)	150 (84%)
Switzerland FOPH Facebook	345 in multiple languages	345 (100%) in multiple languages	113 (100% posts in French) posts in French
Switzerland FOPH Instagram	216	172 (79%)	149 (87%)
Switzerland FOPH Twitter	2,168 tweets obtained in multiple languages	548 (25%) tweets in multipe languages	150 posts in French
UK NHSUK Facebook	621	172 (27.7%)	150 (87%)
UK NHSUK Instagram	45	45 (100%)	44 (98%)
UK NHSUK Twitter	1,695	272 (16%)	150 (55%)

For each country, we selected its public health organisation’s Facebook and Twitter accounts, and, when available, its Instagram page. When more than one health organisation was involved in the COVID-19 communication campaign, we selected either two organisations or the one with the greatest number of social media followers. For Sweden, our analysis included the PHAS’s Facebook and Twitter accounts. The PHAS has a “national responsibility for public health issues” and “works to ensure that the population is protected against communicable diseases and other health threats” [22]. For Switzerland, we included the FOPH’s Facebook, Instagram and Twitter accounts. The FOPH oversees public health at the federal level and is tasked with carrying out information campaigns during health crises [23]. Unlike Switzerland, the UK had multiple organisations under the NHS and the Department of Health and Social Care that communicated about COVID-19 on social media. We selected the accounts of NHSUK, as it had the greatest number of followers. Similarly, more than one federal health organisation was involved in the dissemination of COVID-19 information in Germany and Norway at the national level. In Germany, our data includes the BMG’s Facebook and Instagram pages, along with the RKI’s Twitter account. The RKI is dedicated to the investigation and prevention of infectious diseases [24]. We were unbale to obtain tweets shared by the BMG. For Norway, we selected the NIPH and NDH’s Facebook and Twitter accounts. Both organisations fall under the Ministry of Health and Care services and have been active in different capacities during the pandemic. Norway’s Corona Commission “pointed out that the primary task of the NIPH is to deliver knowledge, while the NDH is a government body, and uses the knowledge as a basis for its assessments” [25]. Neither of the two organisations posted on Instagram in 2020.

For the thematic part of the analysis, we partly based our categories on the 16 themes that Lwin et al. (2016) had adapted for social media from the CERC model. We deductively adopted 14 of their categories that were not specific to the Zika outbreak and complemented them with 19 posts that were derived inductively during the coding process. Together, they added up to 33 categories in total (see Table 2 for an overview of the categories). Several themes that emerged less than a few times throughout the entire coding process were categorised as “other”. The coding was completed by the authors. Each country was coded by one person, except for Norway which had two coders, and Switzerland and the UK which were coded by one person. It is also important to note that the themes were not mutually exclusive, meaning that each post typically contained more than one theme, sometimes as many as five or six, rather than being limited to a single category. Therefore, the total of the percentages discussed in the results section for each account often surpasses 100%.

**TABLE 2:** Overview of the 33 thematic categories (Geneva, Switzerland. 2022).

Theme Cateogry	Definition	Example from the United Kingdom
(1) Disease Mechanisms	Messages about how COVID-19 functions as a virus	Coronavirus is a respiratory virus which affects the respiratory tract
(2) Symptoms	Posts about symptoms associated with COVID-19	The most common symptoms of #coronavirus are: New continuous cough OR High temperature (37.8°or higher) If you have either of these, you need to…
(3) Recovering From COVID-19	Messages about recovering from COVID-19 or “long-COVID-19”	Covid-19 affects us all. No one is free from risk. Although most people recover without treatment, some people can experience #LongCovid for weeks or even months. If you are suffering from long-term symptoms, speak to your GP or visit
(4) Responders	References to organisations or individuals who are responsible for responding to the emergency	DHSC, PHE and NHS England experts are closely monitoring the outbreak of a novel coronavirus in Wuhan, China
(5) Medical Information	Messages sharing medical information or advice	There have been some news reports about vitamin D reducing the risk of coronavirus. However, there is no evidence that this is the case. Read more about vitamin D on our page
(6) Case Reports	Posts about the number of cases and deaths attributed to COVID-19	Coronavirus update In Northern Ireland, there have been 151 concluded tests, of which 150 were confirmed negative, and 1 was confirmed positive
(7) External Information Sources and Infolines	Posts pointing to websites or sources about COVID-19 external to the organisation, or to infolines	HelplinesNI.com has been updated to include details of helplines that have been set up in response to COVID-19 to support people in Northern Ireland
(8) Countering Misinformation	Messages explicitly meant to counteract misinformation, including “fake news”	If you are contacted by the NHS Test and Trace service, you will never be asked to make any form of payment
(9) Vulnerable Groups	Statements meant specifically for vulnerable people and groups at risk	Extremely vulnerable people who have been “shielding” in England are now able to spend time outdoors while continuing to follow social distancing guidelines
(10) Pregnancy	COVID-19 messages targeting pregnant women	If you are pregnant it is important that you still attend your antenatal appointments and continue to seek advice from your midwife or maternity team
(11) University	Messages targeting university students	Are you a student planning your return home for the holidays? Click here to find out how you can minimise your risk during the festive season
(12) Handwashing	COVID-19 posts about handwashing	Handwashing still plays a key role in protecting yourself and others from coronavirus. For guidance on good handwashing technique, visit our page…
(13) How to Sneeze and/or cough	Posts that discuss how to sneeze/cough responsibly	Cough or sneeze into your elbow, or a tissue, and dispose in a bin
(14) Other and/or Generic Hygiene Recommendations	Messages making other hygiene recommendations, or simply generic hygiene recommendations	Don’t touch your face
(15) Air Circultation	Messages about air flow	In enclosed spaces, COVID-19 hangs in the air like smoke. So open windows to clear it away
(16) Social Distancing	Posts reminding people to socially distance during the COVID-19 pandemic	If you’re planning to go out over the #BankHolidayWeekend, make sure to keep 2 m apart from others
(17) Avoid People	Posts recommending people to not meet others	Christmas is fast approaching, but coronavirus cases across Wales are rising sharply again. To protect ourselves and our loved ones, we must not mix with other people. We all need to take steps to make sure we don’t invite coronavirus into our homes this Christmas
(18) Stay at Home	Messages encouraging people to stay at home as much as possible during the pandemic	Staying at home during #Ramadan will play an important part in the effort to slow the spread of coronavirus (COVID-19)
(19) Face Masks	Posts that discuss face masks	Today is World Contraception Day. Masks are important for reducing the spread of Covid-19, just like condoms are vital for preventing the spread of sexually transmitted infections
(20) Covid-19 Vaccine	Messages about coronavirus vaccines	The Covid-19 vaccine has been through three phases of clinical trials to ensure it meets the highest standards of safety and effectiveness
(21) Quarantine and/or Self-isolation	Statements indicating when it is required to quarantine	You should self-isolate if you develop symptoms
(22) Teleworking	Messages about working from home during the COVID-19 pandemic	Work from home if you can
(23) Government-imposed restrictions and Lockdowns	Posts describing COVID-19-related restrictions and lockdowns	Following a surge in COVID-19 cases, local councils have set out areas of Leicestershire that are included in the localised lockdown
(24) Travel- and Border-related	Information concerning border controls and travel regulations related to COVID-19	Been to Wuhan, China in the last 14 days? Follow the latest advice even if you don’t have any symptoms of coronavirus: 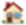 Stay indoors 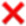 Avoid contact with others where possible 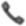 Call NHS 111 Find out more
(25) Contact Tracing	Messages communication about contact tracing	The NHS Test and Trace service launches tomorrow (Thursday 28 May). You can play your part to help control the virus and get life back to normal. Here’s what we need YOU to do
(26) Testing	Posts about COVID-19 testing	Anyone aged 5 or over who has coronavirus symptoms is now eligible for a test
(27) Children and/or Schools	COVID-19 message that are about children and/or schools	Children aged under 5-years-old with symptoms of coronavirus are now eligible for testing in Northern Ireland
(28) Religious and Non-Religious Occasions	Messages developed for religious and non-religious occasions. (But not Christmas period.)	Thank you for not putting yourselves or loved ones at risk this Halloween. Monster mash at home and find other creative ways to have fun safely
(29) Christmas-related	Messages about the Christmas/holiday period related to COVID-19	This year we all face difficult choices and we know that Christmas is going to look very different. With C-19 cases in practically every neighbourhood and a new variant spreading rapidly in parts of the country, it is vital that everyone puts safety first.
(29) Mental Support	Posts designed to help people cope with the impact of the pandemic.	Looking after your mental health while you stay at home. Take 5 steps to wellbeing
(30) Exercise	Messages about exercising as it relates to COVID-19 and the pandemic	Exercise daily to keep your mind and body in good health. - You can leave home once a day to exercise. - If you’re thinking of going for a run this morning make sure to stay 2 m away from others
(31) Gratitude	Messages thanking individuals/groups/stakeholders for their role during the pandemic	Clap for our key workers
(32) Common Responsibilityand Solidarity	Messages encouraging solidarity in the battle against COVID-19	You can play your part in stopping the spread of #coronavirus
(33) Other	Other messages related to COVID-19	Example of Survey: We want to know how easy it is to find and understand our information about getting a coronavirus test. You can help by filling out this survey. We’ll ask you to look at a website page and find and read the information there. https://nhsdigital.eu.qualtrics.com/jfe/form/SV_6g0N7tcCTMmn9qd

Given the number of languages involved, we were unable to perform an inter-coding test; and relying on an online translator would have prevented us from understanding country- and context-specific words and phrases. We also analysed the multimedia (photos and videos) that accompanied each post, which would have posed additional translation challenges. During two practice coding sessions, we coded a small sample of UK social media posts to ensure that differences in interpretation were discussed. In case of any doubts during the coding process, the coders sought the opinion of the other coders during the handover session.

For the first part of the study, which looks at the frequency of COVID-19 social media posts, we divided 2020 by month. Whereas for the thematic part, we split the year into four periods to facilitate the analysis (see Table 3 for an overview of the thematic parts’ temporal breakdown).

**TABLE 3:** Temporal breakdown of 2020 for thematic analysis (Geneva, Switzerland. 2022).

(1) Pre-pandemic (1 January–11 March 2020)	(2) First Lockdowns (11 March–10 May 2020)
The first period, “Pre-pandemic”, spans from 1 January 2020 to 11 March 2020, at which point the WHO categorised the COVID-19 outbreak as a global pandemic	The second period, “First Lockdown”, covers Germany, Switzerland, Norway and the UK’s first national lockdowns, and ends on 10 May 2020 when Boris Johnson outlined a “conditional plan” to reopen the United Kingdom [36]. By then, Switzerland had been easing its restrictions for more than 2 weeks [37]. The UK was further behind, having extended their lockdown by at least 3 weeks on 16 April 2020 [38]. In Germany, a “contact ban” which prohibited gatherings of more than two people was introduced on 22 March 2020 and lasted until early May 2020 [39]. Norway’s national lockdown started on 12 March 2020 and were partially eased starting the second week of May 2020 [40]. Sweden, which didn’t implement a national lockdown during this period, did, nevertheless, impose new restrictions. For example, gatherings of over 500 people were banned on March 11, 2020, before being reduced to less than 50 people 2 weeks later [42]. Then, on 17 March, colleges and universities, as well as schools for children older than 17, switched to distance learning [43]. The PHAS also issued new guidance for restaurants, bars and coffee shops on 24 March 2020 that restricted queuing and the number of people permitted per table [43]
**(3) Post-lockdowns (11 May–14 September 2020)**	**(4) Return of Restrictions (15 September–31 December 2020)**
The third period, “Post-lockdowns”, begins on 11 May 2020 when Switzerland and Germany had already started to ease some of their restrictions, and ends on 14 September 2020 as the “rule of six” came into force in the United Kingdom, banning social gatherings of more than six people [44]. The UK started only a phased re-opening of schools and non-essential shops in England on 1 June and 15 June 2020, respectively [27]. After introducing a plan to gradually reopen Norway on 10 April 2020, the government began to remove some restrictions in May and June 2020. The Norwegian government allowed gatherings of up to 50 people in public places starting on 7 May 2020, while schools reopened on 11 May 2020. Norwegian bars were allowed to reopen only on 1 June 2020 [33]. Sweden began to remove its restrictions on 1 July 2020 [34]. After these initial differences, the next few months were characterised by an easing of restrictions, although there were local variances. For example, the UK’s first local lockdowns came into force in Leicester and parts of Leicestershire on 4 July 2020, while the German authorities in north-western Rheda-Wiedenbrueck introduced new lockdown measures in late June 2020 in response to a major outbreak in a slaughterhouse [35]	The fourth period, “Return of Restrictions”, covers the remainder of the year and is characterised by the reintroduction of restriction measures in the five countries. In Switzerland, the Federal Council banned spontaneous public gatherings of more than 15 people on 18 October 2020 [36]. In the UK, Boris Johnson announced a second lockdown in England on 31 October 2020 to prevent a “medical and moral disaster” for the NHS [27]. In Norway, the government began to tighten COVID-19 measures in late October 2020 when they restricted gatherings in private houses to five people [37]. On 5 November 2020, they introduced further restrictions and recommended that people stay at home [38]. Finally, in Sweden, the PHAS introduced new guidelines on 19 October 2020, making it possible for additional measures to be introduced at the regional or local level alongside national recommendations [39]. This was followed by a ban on the sale of alcohol after 10 pm in restaurants and bars on 20 November 2020

## Results

Most of the Facebook and Instagram accounts that we analysed communicated almost exclusively about COVID-19 and the pandemic in 2020. Twitter accounts tended to also include messages on other topics, which we excluded from our analysis. For example, Norway’s NIPH devoted 35% of its tweets to non-COVID-19-related communication (see Table 1 for breakdown).

### Posting Frequency

**FIGURE 1:**
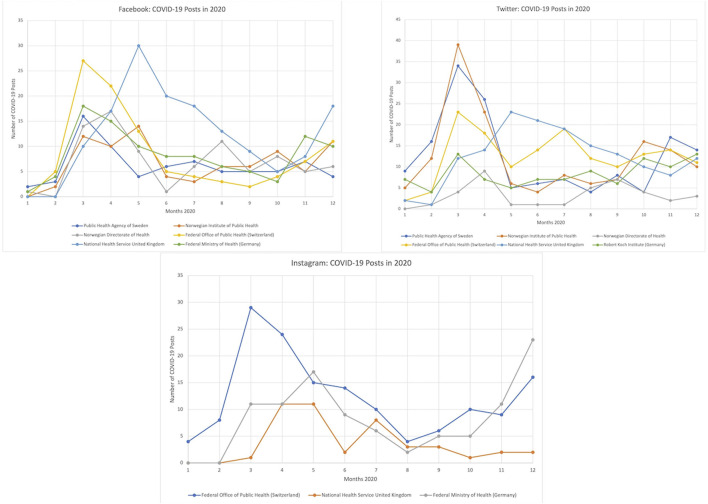
Posting frequency per account (Geneva, Switzerland. 2022).

The rate at which the authorities posted about COVID-19 on social media fluctuated throughout 2020 (see Figure 1). Most accounts began posting about COVID-19 towards the end of February, before notably increasing their communication in March as the “first wave” hit Europe [41]. Each account’s posting frequency peaked between March and May 2020, before dropping considerably during the summer, and then rebounding in the final months of the year during the “second wave” [42].

### Thematic Analysis

The messages that the organisations focused on also varied throughout the year. Overall, across all countries and platforms, the five most frequently communicated themes in 2020 were social distancing (15%), handwashing (14%), case reports (12%), staying at home (10%), and common responsibility (10%) (Table 4 provides an overview of the top 10 most frequently communicated themes in 2020).

**TABLE 4:** Top 10 most frequently communicated themes (Geneva, Switzerland. 2022).

Themes	Period 2020	Germany			Norway				Sweden		Switzerland			United Kingdom			Across All Platforms and Countries in 2020
		BMG Facebook	BMG Instagram	RKI Twitter	NDH Facebook	NDH Twitter	NIPH Facebook	NIPH Twitter	PHAS Facebook	PHAS Twitter	FOPH Facebook	FOPH Instagram	FOPH Twitter	NHS UK Facebook	NHS UK Instagram	NHS UK Twitter	
Case Reports	Pre-pandemic (1 January–11 March 2020)	1 (10	0 (0%)	3 (23%)	0 (0%)	0 (0%)	2 (33%)	9 (26%)	2 (22%)	6 (17%)	0 (0%)	2 (9%)	7 (40%)	0 (0%)	0 (0%)	1 (11%)	33 (18%)
	First Lockdowns (12 March–11 May 2020)	0 (0%)	0 (0%)	5 (45%)	0 (0%)	0 (0%)	1 (5%)	8 (17%)	0 (0%)	5 (10%)	1 (7%)	0 (0%)	12 (36%)	0 (0%)	0 (0%)	0 (0%)	32 (7%)
	Post-lockdowns (12 May–14 September 2020)	8 (32%)	2 (8%)	11 (31%)	0 (0%)	0 (0%)	0 (0%)	3 (13%)	0 (0%)	4 (17%)	0 (0%)	0 (0%)	28 (54%)	0 (0%)	0 (0%)	0 (0%)	56 (11%)
	Return of Restrictions (15 September–31 December 2020)	2 (6%)	4 (10%)	3 (8%)	0 (0%)	0 (0%)	0 (0%)	8 (18%)	0 (0%)	5 (12%)	0 (0%)	2 (5%)	29 (63%)	0 (0%)	0 (0%)	1 (3%)	54 (11%)
COVID-19 Press Conferences	Pre-pandemic (1 January–11 March 2020)	0 (0%)	0 (0%)	2 (15%)	0 (0%)	3 (100%)	0 (0%)	7 (21%)	0 (0%)	8 (23%)	0 (0%)	0 (0%)	6 (31%)	0 (0%)	0 (0%)	0 (0%)	26 (14%)
	First Lockdowns (12 March–11 May 2020)	0 (0%)	0 (0%)	4 (36%)	0 (0%)	0 (0%)	0 (0%)	0 (0%)	0 (0%)	24 (47%)	0 (0%)	0 (0%)	10 (30%)	0 (0%)	0 (0%)	0 (0%)	38 (8%)
	Post-lockdowns (12 May–14 September 2020)	0 (0%)	0 (0%)	9 (25%)	0 (0%)	0 (0%)	0 (0%)	0 (0%)	0 (0%)	1 (4%)	0 (0%)	0 (0%)	6 (11%)	1 (1%)	0 (0%)	0 (0%)	16 (3%)
	Return of Restrictions (15 September–31 December 2020)	0 (0%)	0 (0%)	1 (27%)	0 (0%)	0 (0%)	0 (0%)	1 (2%)	0 (0%)	2 (5)	4 (16%)	5 (13%)	4 (9%)	0 (0%)	3 (50%)	0 (0%)	8 (2%)
Social Distancing	Pre-pandemic (1 January–11 March 2020)	1 (10%)	0 (0%)	0 (0%)	0 (0%)	0 (0%)	3 (50%)	0 (0%)	0 (0%)	0 (0%)	3 (20%)	3 (14%)	1 (5%)	1 (33%)	0 (0%)	0 (0%)	12 (7%)
	First Lockdowns (12 March–11 May 2020)	4 (12%)	2 (6%)	0 (0%)	18 (58%)	0 (0%)	7 (32%)	0 (0%)	7 (29%)	1 (2%)	8 (18%)	11 (23%)	0 (0%)	5 (18%)	10 (67%)	9 (25%)	82 (17%)
	Post-lockdowns (12 May–14 September 2020)	11 (44%)	12 (50%)	3 (8%)	14 (52%)	1 (8%)	8 (33%)	0 (0%)	15 (71%)	2 (9%)	0 (0%)	9 (22%)	0 (0%)	8 (10%)	3 (13%)	12 (18%)	98 (21%)
	Return of Restrictions (15 September–31 December 2020)	8 (26%)	13 (35%)	7 (19%)	3 (14%)	1 (8%)	5 (17%)	0 (0%)	11 (55%)	0 (0%)	3 (13%)	7 (18%)	0 (0%)	4 (9%)	2 (33%)	3 (8%)	67 (14%)
Staying at Home	Pre-pandemic (1 January–11 March 2020)	0 (0%)	0 (0%)	0 (0%)	0 (0%)	0 (0%)	2 (33%)	0 (0%)	3 (33%)	0 (0%)	1 (7%)	6 (27%)	0 (0%)	0 (0%)	0 (0%)	0 (0%)	12 (7%)
	First Lockdowns (12 March–11 May 2020)	6 (18%)	10 (29%)	1 (9%)	11 (29%)	0 (0%)	2 (9%)	0 (0%)	6 (25%)	0 (0%)	16 (36%)	17 (35%)	3 (9%)	11 (39%)	0 (0%)	11 (30%)	94 (20%)
	Post-lockdowns (12 May–14 September 2020)	1 (4%)	0 (0%)	0 (0%)	12 (44%)	0 (0%)	7 (29%)	0 (0%)	7 (33%)	1 (2%)	0 (0%)	2 (24%)	0 (0%)	2 (3%)	1 (4%)	3 (4%)	36 (7%)
	Return of Restrictions (15 September–31 December 2020)	1 (3%)	3 (7%)	3 (8%)	3 (14%)	0 (0%)	1 (3%)	0 (0%)	6 (30%)	0 (0%)	3 (13%)	4 (11%)	0 (0%)	0 (0%)	0 (0%)	2 (3%)	26 (5%)
Handwashing	Pre-pandemic (1 January–11 March 2020)	2 (20%)	0 (0%)	1 (8%)	1 (33%)	0 (0%)	4 (18%)	0 (0%)	2 (8%)	0 (0%)	5 (33%)	5 (23%)	0 (0%)	2 (67%)	0 (0%)	3 (33%)	25 (14%)
	First Lockdowns (12 March–11 May 2020)	2 (6%)	4 (12%)	0 (0%)	18 (58%)	1 (8%)	8 (36%)	0 (0%)	3 (13%)	0 (0%)	0 (0%)	5 (10%)	1 (3%)	1 (4%)	0 (0%)	3 (8%)	46 (10%)
	Post-lockdowns (12 May–14 September 2020)	11 (44%)	12 (50%)	3 (8%)	11 (41%)	0 (0%)	9 (38%)	0 (0%)	3 (14%)	1 (4%)	3 (16%)	4 (10%)	0 (0%)	15 (19%)	4 (17%)	15 (22%)	90 (18%)
	Return of Restrictions (15 September–31 December 2020)	9 (29%)	12 (29%)	7 (19%)	2 (9%)	0 (0%)	4 (13%)	0 (0%)	7 (35%)	0 (0%)	2 (8%)	6 (16%)	1 (2%)	3 (8%)	1 (17%)	2 (5%)	56 (12%)
Face Mask	Pre-pandemic (1 January–11 March 2020)	0 (0%)	0 (0%)	0 (0%)	0 (0%)	0 (0%)	4 (67%)	0 (0%)	0 (0%)	0 (0%)	2 (13%)	3 (14%)	0 (0%)	1 (33%)	0 (0%)	0 (0%)	6 (3%)
	First Lockdowns (12 March–11 May 2020)	4 (12%)	3 (9%)	0 (0%)	0 (0%)	1 (8%)	8 (36%)	0 (0%)	0 (0%)	0 (0%)	3 (7%)	2 (4%)	0 (0%)	0 (0%)	0 (0%)	0 (0%)	15 (3%)
	Post-lockdowns (12 May–14 September 2020)	11 (44%)	11 (46%)	4 (11%)	2 (7%)	3 (30%)	9 (38%)	0 (0%)	0 (0%)	0 (0%)	2 (4%)	7 (17%)	0 (0%)	19 (24%)	4 (17%)	14 (21%)	82 (17%)
	Return of Restrictions (15 September–31 December 2020)	10 (32%)	13 (31%)	7 (19%)	0 (0%)	0 (0%)	4 (13%)	0 (0%)	1 (11%)	0 (0%)	3 (13%)	7 (18%)	0 (0%)	3 (8%)	2 (33%)	2 (5%)	50 (10%)
COVID-19 Vaccines	Pre-pandemic (1 January–11 March 2020)	0 (0%)	0 (0%)	0 (0%)	0 (0%)	0 (0%)	1 (17%)	0 (0%)	0 (0%)	0 (0%)	0 (0%)	3 (14%)	0 (0%)	0 (0%)	0 (0%)	0 (0%)	3 (2%)
	First Lockdowns (12 March–11 May 2020)	1 (3%)	2 (6%)	1 (9%)	0 (0%)	0 (0%)	0 (0%)	0 (0%)	0 (0%)	0 (0%)	0 (0%)	2 (4%)	0 (0%)	0 (0%)	0 (0%)	0 (0%)	4 (1%)
	Post-lockdowns (12 May–14 September 2020)	0 (0%)	2 (8%)	3 (8%)	0 (0%)	0 (0%)	0 (0%)	0 (0%)	0 (0%)	2 (9%)	0 (0%)	9 (22%)	0 (0%)	1 (1%)	0 (0%)	2 (3%)	10 (2%)
	Return of Restrictions (15 September–31 December 2020)	4 (16%)	11 (26%)	8 (22%)	0 (0%)	0 (0%)	4 (13%)	2 (5%)	1 (5%)	3 (7%)	4 (17%)	7 (18%)	2 (4%)	12 (32%)	3 (50%)	8 (21%)	67 (14%)
Common Responsibility and Solidarity	Pre-pandemic (1 January–11 March 2020)	1 (10%)	0 (0%)	1 (8%)	0 (0%)	0 (0%)	0 (0%)	1 (3%)	0 (0%)	2 (6%)	0 (0%)	1 (5%)	0 (0%)	0 (0%)	0 (0%)	0 (0%)	6 (3%)
	First Lockdowns (12 March–11 May 2020)	12 (35%)	12 (35%)	1 (9%)	8 (26%)	0 (0%)	3 (14%)	0 (0%)	14 (58%)	1 (2%)	11 (24%)	11 (23%)	0 (0%)	4 (14%)	2 (13%)	1 (3%)	70 (15%)
	Post-lockdowns (12 May–14 September 2020)	5 (20%)	2 (8%)	4 (11%)	1 (4%)	0 (0%)	0 (0%)	0 (0%)	12 (57%)	0 (0%)	3 (16%)	0 (0%)	0 (0%)	5 (6%)	0 (0%)	3 (4%)	54 (10%)
	Return of Restrictions (15 September–31 December 2020)	3 (10%)	2 (5%)	5 (14%)	1 (5%)	0 (0%)	2 (7%)	6 (14%)	7 (35%)	3 (7%)	6 (24%)	2 (5%)	1 (2%)	0 (0%)	0 (0%)	2 (5%)	54 (11%)
Symptoms	Pre-pandemic (1 January–11 March 2020)	4 (40%)	0 (0%)	2 (15%)	2 (6%)	0 (0%)	0 (0%)	0 (0%)	4 (44%)	1 (3%)	1 (7%)	7 (32%)	1 (5%)	1 (33%)	0 (0%)	0 (0%)	18 (10%)
	First Lockdowns (12 March–11 May 2020)	2 (6%)	0 (0%)	1 (9%)	1 (32%)	0 (0%)	0 (0%)	1 (2%)	2 (8%)	0 (0%)	4 (9%)	1 (2%)	2 (6%)	3 (10%)	1 (7%)	6 (17%)	22 (5%)
	Post-lockdowns (12 May–14 September 2020)	2 (8%)	6 (25%)	1 (28%)	0 (0%)	0 (0%)	0 (0%)	0 (0%)	0 (0%)	0 (0%)	4 (21%)	5 (12%)	0 (0%)	6 (8%)	2 (9%)	7 (10%)	34 (7%)
	Return of Restrictions (15 September–31 December 2020)	3 (10%)	9 (21%)	0 (0%)	1 (4%)	0 (0%)	0 (0%)	0 (0%)	0 (0%)	0 (0%)	4 (17%)	4 (11%)	0 (0%)	3 (8%)	0 (0%)	2 (5%)	34 7%)
External Sources of Information and Hotlines	Pre-pandemic (1 January–11 March 2020)	0 (0%)	0 (0%)	1 (8%)	0 (0%)	0 (0%)	3 (50%)	5 (15%)	2 (22%)	1 (3%)	2 (13%)	9 (40%)	0 (0%)	1 (33%)	0 (0%)	2 (22%)	26 (14%)
	First Lockdowns (12 March–11 May 2020)	0 (0%)	0 (0%)	0 (0%)	3 (10%)	6 (50%)	4 (18%)	8 (17%)	2 (8%)	4 (8%)	1 (4%)	1 (2	0 (0%)	1 (4%)	1 (7%)	0 (0%)	29 (6%)
	Post-lockdowns (12 May–14 September 2020)	0 (0%)	0 (0%)	1 (28%)	1 (4%)	3 (30%)	3 (13%)	5 (21%)	0 (0%)	1 (4%)	0 (0%)	2 (5%)	0 (0%)	1 (1%)	0 (0%)	0 (0%)	17 (3%)
	Return of Restrictions (15 September–31 December 2020)	0 (0%)	0 (0%)	1 (27%)	4 (18%)	3 (25%)	6 (20%)	4 (9%)	0 (0%)	2 (5%)	0 (0%)	0 (0%)	0 (0%)	0 (0%)	1 (17%)	0 (0%)	21 (4%)

#### Period 1: Pre-Pandemic (1 January–11 March 2020)

Across all countries and platforms, the five most frequently communicated themes during the pre-pandemic period were case reports (18%), handwashing (14%), press conferences (14%), external sources of information (14%), and symptoms (10%). In addition to these messages, an emphasis on the need to stay at home, which was featured in 7% of posts across all countries and platforms, began to emerge. Some countries, such as Switzerland (27% on Instagram) and Sweden (33% on Facebook), put a greater emphasis on this message. However, it is important to note that this period’s sample size is relatively small. For example, Sweden’s PHAS—for which we coded all COVID-19-related Facebook messages from 2020—shared only nine COVID-19 messages on Facebook prior to 11 March 2020. The UK accounts also shared a limited number of messages—12 posts across all three platforms in our dataset in total—during the pre-pandemic period.

The most shared Swiss message on Instagram from this period was the COVID-19 information hotline set up by the FOPH. It was included in 41% of the FOPH’s Instagram posts—for which we analysed 79% of its posts from 2020—but was only found three more times in our dataset in the remainder of the year.

On Twitter, most organisations prioritised different messages compared with Facebook and Instagram. Switzerland’s FOPH focused less on handwashing and stay-at-home measures on Twitter and instead prioritised tweets that reported on the number of cases/deaths in Switzerland (40%). Sweden’s PHAS also stressed different themes on Twitter—such as press conferences (23%), case reports (17%) and first responders (11%)—compared to Facebook. In Norway, while the two organisations were relatively inactive on Facebook, they diverged in their Twitter communication during the pre-pandemic period. The NDH only tweeted about press conferences (100%), whereas the NIPH had a more active and diverse communication, tweeting about case reports (26%), press conferences (21%), as well as quarantine and isolation measures (6%) and disease mechanisms (6%). During this period, German organisations also differed in some of the messages that they prioritised on Facebook and Twitter. Germany’s RKI included case reports and press conferences in 23% and 15% of their tweets, respectively. These two messages were not featured on the BMG’s Facebook page. However, both accounts posted about symptoms (Facebook: 40%; Twitter: 15%), first responders (Facebook: 60%; Twitter: 23%) and medical recommendations (Facebook: 30%; Twitter: 30%).

#### Period 2: First Lockdowns (12 March–10 May 2020)

During the lockdown period, health authorities, unsurprisingly, prioritised messages about staying at home and social distancing. Across all countries and platforms, the five most frequently communicated themes were staying at home (20%), social distancing (17%), common responsibility (15%), handwashing (10%), and press conferences (8%).

NHSUK put a strong emphasis on stay-at-home messages (Facebook: 39%; Twitter 30%). Switzerland’s FOPH also discussed stay-at-home measures in over a third of its Facebook and Instagram messages. It also communicated about the public’s common responsibility in nearly a quarter of their Facebook and Instagram posts.

Similarly, Norway’s NDH communicated about staying at home in 29% of its Facebook posts, alongside messages about social distancing (58%) and handwashing (58%). The NIPH, which was less active Facebook, focused on handwashing (36%), social distancing (32%) and external sources of information (18%). On Twitter, the NIPH tweeted different themes, such as contact tracing (23%), case reports (17%) and testing (10%), while the NDH tweeted mainly about external sources of information (50%) and face masks (25%).

Germany’s BMG, on the other hand, posted about stay-at-home measures in 18% of its Facebook messages. It rather prioritised messages that thanked the public for following COVID-19-related measures (35%) and/or highlighted the public’s common responsibility (35%). However, the BMG also began to disseminate information about COVID-19 on Instagram, where stay-at-home posts (29%) were given a higher priority compared to the average for this period, alongside messages about the public’s common responsibility (35%) and COVID-19’s disease mechanism (21%). In contrast to the other accounts, the RKI’s Twitter output dropped during this period and was largely limited to tweets about COVID-19 case reports (45%) and press conferences (36%).

Although Sweden did not institute an official lockdown during this period, the PHAS’s Facebook communication discussed social distancing and staying at home in 29% and 25% of its Facebook posts, respectively. The PHAS’ Facebook account also put a stronger emphasis on the public’s common responsibility (58%) compared with other accounts during this period.

#### Period 3: Post-Lockdowns (11 May–14 September 2020)

New themes emerged in the third period as less priority was given to stay-at-home messages. Across all countries and platforms, the five most frequently communicated themes were social distancing (21%), handwashing (18%), face masks (17%), case reports (11%), and common responsibility (10%).

In Germany, masks took a central role in the BMG’s Facebook (44%) and Instagram (46%) communication, while the RKI included it in only 11% of its tweets. NHSUK had the second highest rate of messages about face masks (Facebook: 24%, Instagram: 17%; Twitter: 21%).

In Switzerland, the FOPH discussed face masks in just 17% of its Instagam posts. Rather, the FOPH put an above-average emphasis on contact tracing (Facebook: 21%; Instagram: 24%). But the FOPH rarely posted about contact tracing in the second half of the year, as 82% of their Facebook and Instagram posts on this measure were shared in May and June 2020. Norway’s NIPH is one exception to this trend. Their Twitter account put a greater emphasis on contact tracing during the second (23%) and fourth periods (25%) compared to the third (8%).

The Norwegian organisations also communicated less frequently about face masks. The NDH’s Twitter account had the highest ratio of messages about face masks (30%); however, it should be noted that their Twitter communication was limited during this period (only 10 tweets—all posts were coded). The NIPH highlighted travel restrictions (33%) and case reports (11%) in its tweets. On Facebook, the NIPH continued to post about handwashing (38%), social distancing (33%) and stay at home (29%). The NDH also shared similar messages on Facebook.

In Sweden, the PHAS did not communicate about face masks on Facebook nor Twitter. The PHAS’s Facebook page rather focused on social distancing (71%), the public’s common responsibility (57%) and staying at home (33%).

#### Period 4: Return of Restrictions (15 September–31 December 2020)

In autumn 2020, the five countries began to record a resurgence in COVID-19 cases, which led to the reintroduction of certain measures. At the same, they also launched their COVID-19 vaccination campaigns towards the end of the year. Across all countries and platforms, the five most frequently communicated themes during this period were COVID-19 vaccines (14%), social distancing (14%), handwashing (12%), case reports (11%), and face masks (10%).

In the UK, NHSUK devoted a higher percentage of its communication to COVID-19 vaccines (Facebook: 32%; Instagram: 50%; Twitter: 21%) than the other countries. Germany’s BMG also put an above-average focus on COVID-19 vaccine messages on Instagram (26%). In Switzerland, the FOPH focused less on COVID-19 vaccines (Facebook: 17%; Instagram: 18%; Twitter: 4%). Most of the Swiss vaccine messages were shared in December 2020. In the case of Norway, nonly the NIPH discussed COVID-19 vaccines (Facebook: 13%; Twitter: 5%), while in Sweden, the PHAS’s vaccine posts were featured in 7% and 5% of their Twitter and Facebook posts, respectively.

Beyond vaccines, Germany’s BMG and RKI continued to highlight social distancing (Facebook: 26%; Instagram: 35%; Twitter: 19%). Sweden’s PHAS also carried on with its focus on social distancing (55%) on Facebook. In Norway, the NIPH’s Facebook page also highlighted social distancing (17%). Between the UK and Switzerland, the FOPH’s Facebook and Instagram pages prioritised social distancing the most at 13% and 11%, respectively.

Face masks were another main priority of Germany’s BMG (Facebook: 32%; Instagram: 31%; Twitter: 19%). Switzerland’s FOPH continued to post about face masks (18%) relatively frequently on Instagram. Norway and Sweden paid relatively little attention to face masks during this period in their social media posts. Norway’s NIPH put a rather strong emphasis on contact tracing (25%), alongside case reports (18%) and the public’s common responsibility (14%). Sweden’s PHAS also continued to communicate about the public’s common responsibility (Facebook: 35%) and restrictions/lockdowns (Facebook: 35%).

## Discussion

In this study, we have analysed what health authorities from each of the five countries communicated about COVID-19 on social media in 2020. Most health organisations included in this study had a limited presence on Facebook and—especially—Instagram prior to the pandemic. The literature, however, highlights the “importance of making friends before you need them,” as an “organisation should establish itself on social media before the risk and/or crisis situation arises, and demonstrate that the organisation is there to disseminate information, communicate and listen” [43].

As illustrated in Figure 1, the rate at which the accounts posted fluctuated throughout the year. After peaking between March and May 2020, the number of posts per month decreased considerably during the summer. Then, as the second wave surfaced in autumn 2020, most health authorities increased their social media communication once again. Lwin et al. (2018) observed a similar trend in their study of Singaporean health authority’s Facebook communication during the 2016 Zika outbreak [11]. Given the ongoing status of the COVID-19 pandemic at the time, it could be argued that the health authorities overlooked communication on social media during the summer months. For example, once face masks became mandatory on public transportation in Switzerland on 20 July 2020, the FOPH—for which our dataset includes all French Facebook messages from 2020—shared only six French Facebook posts in total until the end of September 2020.

Additionally, accounts from all five countries not only shared comparable posting trends, but they also prioritised similar themes. On Facebook, each account had at least three of the following messages in their top five most frequently communicated themes: handwashing, social distancing, staying at home, face masks, and/or common responsibility. On Twitter, the top two most frequently communicated themes in Germany, Sweden and Switzerland were case reports and press conferences. In the case of Norway, case reports were the NIPH’s most tweeted theme, while press conferences were its fifth. The NDH prioritised these two categories even less. NHSUK was the clear odd one out, their top five most frequently shared categories did not include press conferences nor case reports, and rather resembled their Facebook and Instagram communication.

Each of the five countries also covered the COVID-19 preventive measures recommended by the WHO [4]—including “air circulation”, which was featured less prominently (5% of total messages)—in their Facebook communication. Norway and Sweden also had a relatively limited percentage of messages about “sneezing and coughing responsibly”, while Germany’s BMG put a relatively greater focus on this theme by featuring it in 46% of their Facebook posts during the third period. Some of the communication that were unique to an organisation include NHSUK’s limited number of posts targeting pregnant women and Sweden’s posts about exercising during the pandemic (Facebook: 13% during the second period).

Overall, the authorities’ social media communication included the majority of the CERC’s recommendations that Lwin et al. (2018) had adapted for risk communication on social media. On Facebook, each account had communicated at least three quarters of Lwin et al.’s categories in 2020. Yet, as most accounts only started to post regularly about COVID-19 in late February 2020, the authorities could be criticised for not having sufficiently communicated with the public during the “pre-pandemic” period. They did, nevertheless, share most of the messages suggested for this period on some of their accounts at a relatively limited frequency. For example, they posted a limited number of messages that recognised the emerging risk of COVID-19 and promoted behavioural changes [11].

Most organisations prioritised different messages on Twitter than on Facebook or Instagram. The heightened attention on case reports and press conferences on Twitter by some accounts could be because the platform is above all else used to follow the news. Therefore, the Swiss and Swedish health authorities could have decided that a different communication plan would be more suitable for Twitter. The literature also demonstrates that journalists have integrated Twitter into their daily news production practices [44]. The FOPH’s messages on the number of new cases were typically accompanied by their latest reports, which could contain information that is relevant for journalists.

As the second least active account, NHSUK’s Instagram page—for which we analysed all messages from 2020—shared only 44 posts related to COVID-19 on Instagram in 2020. This is interesting to note since the account’s number of followers increased by tenfold in 2020, reaching over 390,000 followers by the end of the year. In a rare study on Instagram, researchers examined the social media communication of the WHO, the CDC and Doctors Without Borders (MSF) during the 2014 Ebola crisis [44]. The photo-sharing platform was included by the researchers even though it was still an “emerging” social media platform in 2014 [44]. Their content analysis yielded differences between the organisations and platforms. Most notably, they showed that Instagram posts generated more interactions than tweets, on average, which could be viewed as “indicative of an attentive and responsive audience” (45). These findings, coupled with Instagram’s potential for two-way communication, pushed the authors to recommend that while it is important to maintain a presence on most social media platforms, Instagram could be prioritised given than it may yield the greatest returns.

### Limitations

One of the study’s limitations is that it does not analyse Instagram “stories” and “paid posts”, limiting the study to Instagram “posts”. Concerning our sample, it does not include all social media posts shared by the seven organisations in 2020. That said, we had reached a point of saturation during the coding process. Our sample also does not include Instagram posts from Norway and Sweden, even though the latter did communicate on Instagram about COVID-19 through other accounts. We were also unable to collect data from the German BMG’s Twitter account, which would have permitted for an intra-platform and intra-organisation comparison. Finally, considering that each of the five countries had different COVID-19 outbreak and policy timelines, it is possible that our breakdown of 2020 into four periods for the thematic analysis might have not suited every country equally.

### Conclusion

To the best of our knowledge, this is the first study to compare the social media risk communication of Germany, Norway, Sweden, Switzerland, and the United Kingdom. As such, our study provides a comparative lens to their key thematic areas and frequency of communication, which may be useful for monitoring and reviewing public health communication strategies. We found that the authorities’ communication frequency fluctuated throughout 2020, with the summer months leading to a decrease in communication. Overall, the authorities’ communication included the majority of the CERC’s recommendations, as well as the COVID-19 preventive measures suggested by the WHO. Our comparison, however, also highlighted messages—such as long COVID, pregnancy and exercising during the pandemic—that could have featured more prominently in a targeted COVID-19 campaign. Our results did show, nevertheless, that some of the health organisations favoured different messages on Twitter compared to Facebook and Instagram, potentially suggesting that they prioritised different audiences and objectives on each of the platforms. Future studies could focus on how the public reacted to different messages on each of the platforms, while also ensuring that emerging, popular social media platforms (such as TikTok) are not left behind. As more individuals now follow health authorities on social media, platforms such as Instagram could hold great potential for future risk communication campaigns and strategies.
